# Treatment of temporomandibular myofascial pain with deep dry needling

**DOI:** 10.4317/medoral.17822

**Published:** 2012-05-01

**Authors:** Luis M. Gonzalez-Perez, Pedro Infante-Cossio, Mercedes Granados-Nuñez, Francisco J. Urresti-Lopez

**Affiliations:** 1 Facultativo Especialista, Servicio de Cirugía Oral y Maxilofacial, Hospital Universitario Virgen del Rocío, Sevilla; 2 Profesor Titular Vinculado, Servicio de Cirugía Oral y Maxilofacial, Hospital Universitario Virgen del Rocío, Universidad de Sevilla; 3 Máster en Cirugía Bucal, Facultad de Odontología, Universidad de Sevilla; 4 Fisioterapeuta, Facultad de Enfermería, Fisioterapia y Podología, Universidad de Sevilla

## Abstract

Objectives: The present study was designed to evaluate the usefulness of deep dry needling in the treatment of temporomandibular myofascial pain. Study Design: We selected 36 patients with myofascial pain located in the external pterygoid muscle (30 women/6 men, mean age=27 years with SD±6,5). We studied differences in pain with a visual analog scale and range of mandibular movements before and after intervention. Results: We found a statistically significant relationship (p<0,01) between therapeutic intervention and the improvement of pain and jaw movements, which continued up to 6 months after treatment. Pain reduction was greater the higher was the intensity of pain at baseline. Conclusions: Although further studies are needed, our findings suggest that deep dry needling in the trigger point in the external pterygoid muscle can be effective in the management of patients with myofascial pain located in that muscle.

** Key words:**Temporomandibular joint, myofascial pain, external pterygoid muscle, trigger point, deep dry needling.

## Introduction

Temporomandibular joint (TMJ) is a bilateral diarthrosis located between the glenoid fossa-temporal eminence complex and the mandibular condyle. Its involvement is multifactorial, and is commonly known by ambiguous terms such as temporomandibular disorder or TMJ pain-dysfunction syndrome. This condition is highly prevalent, and usually has varied clinical presentations characterized by pain in the preauricular area and alterations in the dynamic of open-close mouth movements. Painful hypersensitivity is often observed in the masticatory muscles as origin of myofascial pain syndrome, being the external pterygoid muscle one of the most frequently affected. External pterygoid muscle (Pterygoideus lateralis) is a short, thick and flattened transversely muscle, located in the pterygomaxillary fossa roof that extends from the pterygoid process and greater wing of sphenoid to the neck of the mandibular condyle, being essential to the protrusive and lateral movements of the mandible (1,2).

Most authors agree that the current treatment of nonspecific myofascial pain is multidisciplinary and must be approached from several perspectives. Myofascial pain must be suspected in patients with pain in masticatory muscles located in the preauricular or mandibular area, frontal or temporal regions, or in the ear, along with the existence of painful trigger points (TPs) on palpation. Usually, it is associated with pain provoked by mandibular function and with limitation of interincisal opening (opening <40 mm) (3-9).

Our study had the purpose of aiding to determine the possible importance of myofascial TPs in temporomandibular pathology, and evaluate the usefulness of the deep dry needling (DDN) in the treatment of myofascial pain of the external pterygoid muscle.

## Material and Methods

We studied 36 patients with myofascial pain located in the external pterygoid muscle consecutively selected in the outpatient clinic of the Oral and Maxillofacial Surgery Department of “Virgen del Rocio” University Hospital of Seville (Spain), between May and September 2008, to compare differences in pain with a visual analog scale and range of mandibular movements before and after application of needles for needling the external pterygoid muscle. Patients were included in a diagnostic protocol based on clinical signs and imaging techniques (panoramic radiograph and MRI). The study received prior approval from the Committee for Research and Clinical Ethics of the Hospital, and informed consent was obtained from all patients before inclusion in the study. We included patients older than 18 years of age with temporomandibular pain for more than six months and moderate limitation in mandibular movement (limitation of interincisal opening <40 mm and passive stretching to force the opening ≥ 5 mm, according to I-b group criteria of the International RDC-TMD Consortium), and with a palpable taut band or hypersensitive nodule in the external pterygoid muscle mass by intraoral palpation. We excluded patients under 18 years and those with TMJ internal derangements with anterior disk displacement, degenerative joint disease (osteoarthritis/osteoarthrosis), history of jaw trauma, vascular diseases, migraine and tension headaches, and history of infectious-inflammatory conditions of odontogenic origin.

**Figure 1:**
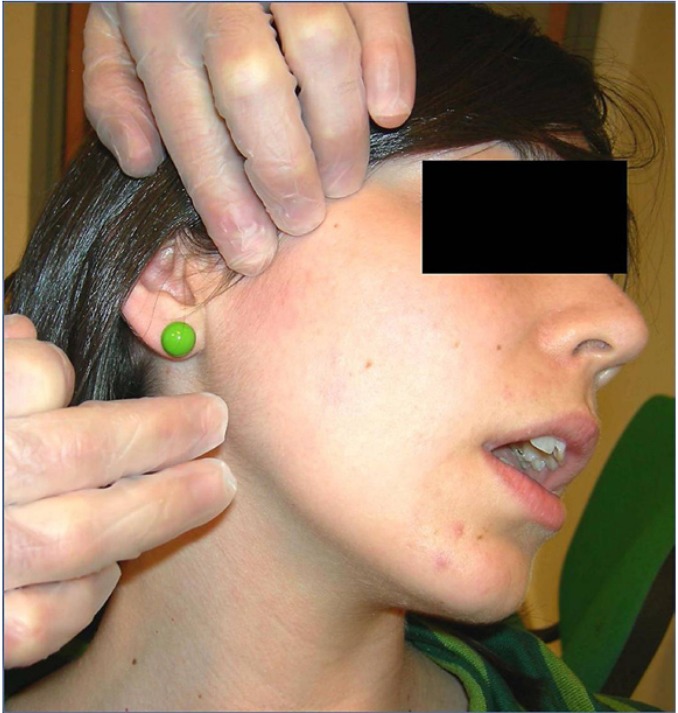
Anatomic location of the external pterygoid muscle for needling.

**Figure 2:**
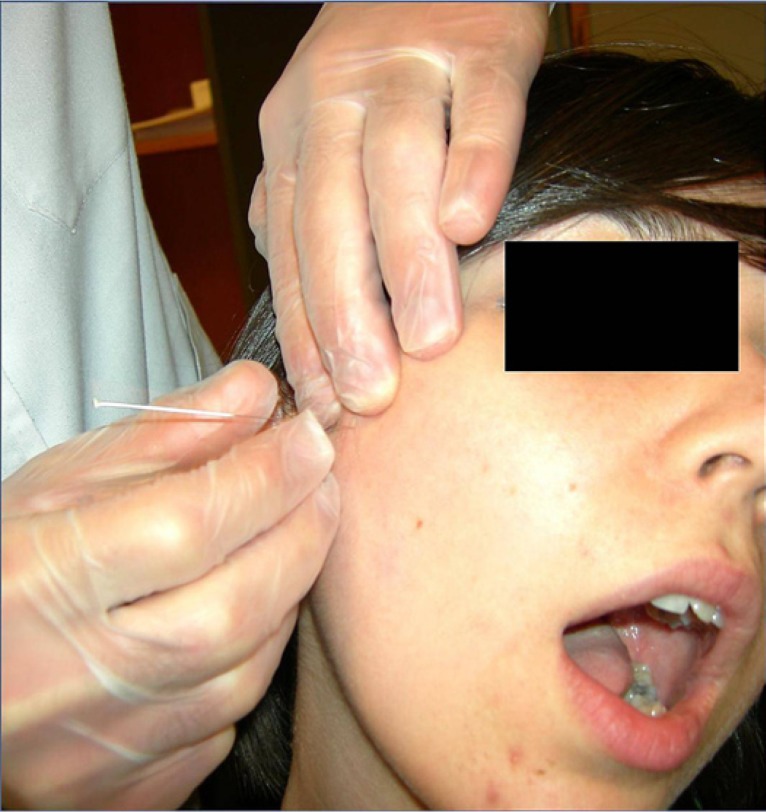
Needling with a 40 mm. sterile needle and removal the plastic guide-tutor, followed by movements of rotation and input-output to enhance the elimination of myofascial pain.

Sterile stainless steel needles with a plastic cylindrical guide, 40 mm in length and 0.25 mm caliber distributed in Spain by Agu-punt®, have been used for the therapy with DDN. A total of 3 sessions were performed per patient at an interval of 1 week, and clinical assessments at 2 weeks, 1 month, 2 months and 6 months after finishing the treatment. The needle was applied after asepsis of the preauricular area with alcohol 90%, manual location of the intra-extraoral mass of the external pterygoid muscle (Fig. 1), uni- or bilaterally, and intramuscular needling (Fig. 2), according to the technique of Koole et al. modified without electromyographic control (10), followed by compressive hemostasis for 1 minute. The needle was inserted perpendicularly through the skin taking into account the relationship between the muscle and TPs with the surrounding anatomical structures. That is why we considered necessary to know the exact location of the TP by palpation and pressure sensivity, and by asking the patient to perform a maximum opening that allowed us to avoid the bony structures that impeded the access to the external pterygoid muscle (coronoid process ahead, mandibular condyle behind, zygomatic arch above, and the sigmoid notch below). A jump or jerk reaction of the muscle (local twitch response) is often produced upon inserting the needle in the TP. If another TP can be detected in any of the other elevator muscles (temporal, masseter and internal pterygoid muscles), these areas were inactivated before DDN of TP in the external pterygoid muscle; so that 4 patients of our study with TPs also in masseter muscle received, in addition to treatment with DDN, an adjuvant treatment with deep tissue massage and manual stretching of the masseter muscle in order to prepare the treatment area (external pterygoid muscle) by muscle relaxation of adjacent structures. The treatment was performed using deep needling in the myofascial TP without the introduction of any substance (dry needling). Four variables were measured: perceived or subjective pain by visual analog scale and range of mandibular movements by Therabite® System rule (mouth opening, laterality and protrusion movements). The signs that were assessed as indicative of the efficacy of DDN were: significant improvement of myofascial pain, recovery of the normal range of mouth opening, incisal straight path during open-close movements, and absence of painful hipersensivity on palpation of external pterygoid muscle with disappearance of the TP. The statistical analysis of results was carried out with SPSS 15.0 software.

## Results

Of the 36 patients studied, 30 (83.3%) were women and 6 (16.6%) men. The mean age was 27 years (SD ± 6.5). Clinical signs were: preauricular pain (in all cases) with a mean value of 8.45 ± 1.46 in visual analog scale, joint noise sounds in mouth opening (in 5 cases), and reduction in the range of mandibular movements (in all cases). Before DDN, mean mandibular movements values were: mouth opening of 2.90 (± 1.10), laterality of 0.20 (± 0.01), and protrusion of 0.1 (± 0.00). Radiographic findings showed no alteration in the morphology of the articular surfaces. MRI studies revealed no significant changes in static and dynamic position of the articular disk in any of the cases studied.

**Figure 3:**
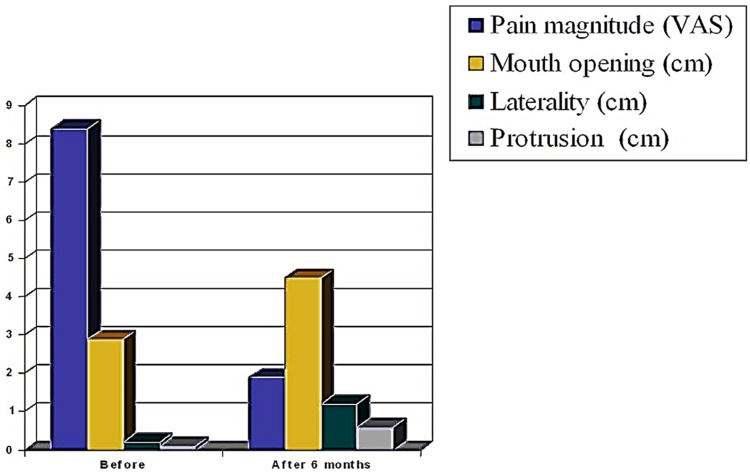
Changes in pain, mouth opening, and protrusive and lateral movements, before and 6 months after DDN in the external pterygoid muscle.

 Table 1 shows the evolution of pain. After treatment, the pain was reduced in 6.5 points (SD ± 1.57). The magnitude of pain reduction was statistically significant (p <0.01) in all controls performed. Mean mandibular movement at 6 months after the DDN were: mouth opening of 4.50 cm (± 0.50), laterality of 1.20 cm (± 0.20), and protrusion of 0.6 cm (±0,01). In (Fig. 3) are represented the changes in pain and functional parameters (opening, lateral and protrusive movements) before and 6 months after DDN.

**Table 1:**
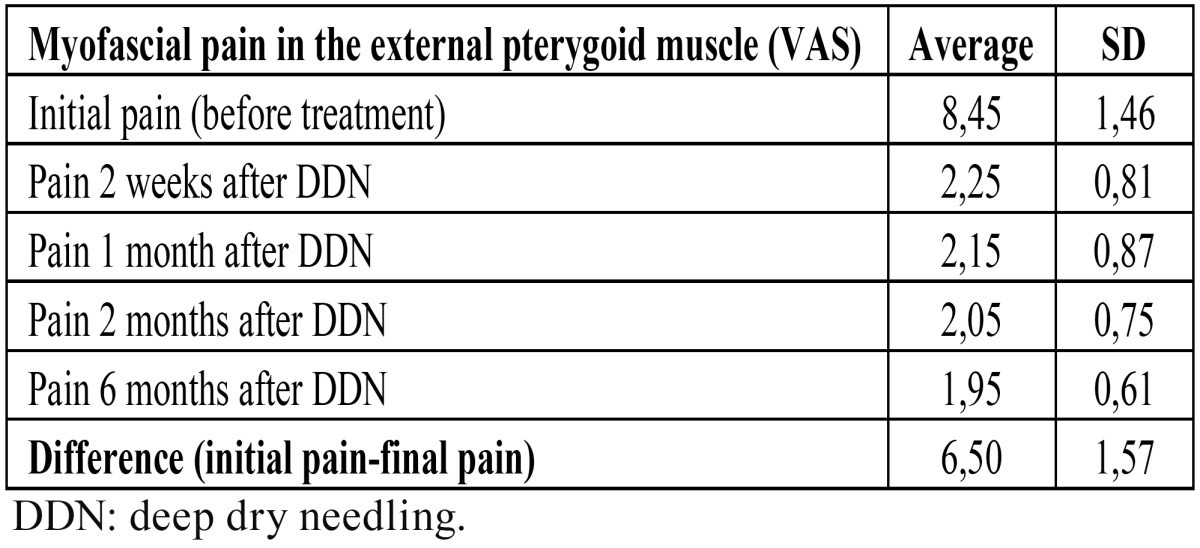
Temporomandibular pain evolution before and after treatment with DDN, assessed by visual analogue scale (VAS).

By analyzing the correlation between pain assessment before the start and the end of treatment, we observed that greatest reductions in pain were achieved in those patients who had high baseline values in visual analog scale before starting treatment. On the contrary, when patients had low values of pain in visual analogue scale, less pain reduction was achieved. The pain response to DDN therapy was observed as early as 2 weeks after DDN, and remained stable without significant changes during the control patient period ( Table 1). Also, the mandibular movement response (opening, laterality and protrusion) was observed in the first control at 2 weeks after DDN, showing no significant variation throughout the control period up to 6 months post-treatment.

## Discussion

Temporomandibular pain of myofascial origin is a condition often referred for evaluation to outpatient clinics of Oral and Maxillofacial Surgery Departments. In more than 90% of cases no underlying cause is found, that is why they are diagnosed as nonspecific or uncomplicated pains, whose treatment is still under study (2,6,11).

Vazquez-Delgado et al. have reviewed the pathophysiologic, clinical and therapeutics aspects of the myofascial pain syndrome (12,13). Usual treatment of temporomandibular myofascial pain in our working environment is a combination of pharmacological and splint therapy, which produces a temporary relief. However, pharmacological treatments soon reach the limit of therapeutic efficacy and they are also associated with side effects (gastrointestinal disorders, drug interactions, and adverse reactions), so that the current trend is the search for alternative treatments (14-16).

Clinical guidelines and protocols about temporomandibular disorders recommend the management of myofascial pain from a multidisciplinary approach (3,4). However, within available treatments for muscle pain, there is a paucity of studies about the effectiveness of TP needling in masticatory muscles, and that is why we set the objective of studying the DDN of myofascial TP in the external pterygoid muscle. The mechanism of inactivation of a TP by needling is unknown, although we consider important the presence of a local twitch response during DDN, because it has a proven relationship with the desired therapeutic effect (6,17). Apparently, the tissular mechanical disruption caused by needle insertion constitutes the specific therapy, but the mechanism of action by which the TP is disabled is unknown. According to Travell, it would be by the mechanical disruption of the self-sustaining mechanism of the TP due to the membrane depolarization of the nerve fibers provoked by the intracellular potassium release and interruption of the central feedback mechanism (1,7). According to Hong, the most reasonable explanation seems to be a neurological mechanism (18), because pain relief after the DDN happens often in few seconds, as we also observed in our study. Probably, local mechanical disruption or reflex central interruption are the most likely mechanisms for breaking the vicious circle of the phenomenon of myofascial TP (11,14,16).

Since by stretching techniques and manual handling is difficult and complicated to access to the external pterygoid muscle, needling of TPs may be necessary. The critical importance of this muscle as origin of temporomandibular myofascial pain makes it worthwhile to develop the necessary skills for invasive treatment by TP needling. The external approach (extraoral DDN) allows the needling of the central TPs in the muscle bellies of the two divisions of the muscle and in the insertional TPs of posterior myotendinous junctions of both divisions. The correct location of the muscle mass is essential for the technique we use, so interference factors represented by the nearby bony structures (zygomatic arch, coronoid process, condyle and sigmoid notch of the mandible) must be eliminated. That is why there is no need to carry out the electromyographic control described in Koole et al. study (10), which is a complicated and uncomfortable technique that we do not think it is necessary in treating outpatients with DDN in which the TP can be detected by careful palpation and pressure sensitivity. Taut bands and TPs in the masseter muscle are other factors to consider as interference, since it can be difficult to recognize the pressure sensitivity on the external pterygoid muscle, which lies in a deeper layer. Masseter taut bands are more superficial and oriented almost perpendicularly to the fibers of the external pterygoid muscle making them easily distinguishable. Hypersensitivity of TPs in the masseter muscle must be inactivated before treatment of TPs in the external pterygoid muscle by DDN, as we did in 4 cases in our study.

In our patients we did not use a intramuscular puncture technique to inject local anesthetics, corticosteroids or botulinum toxin because DDN is just as effective in the myofascial pain caused by TPs in the pterygoid muscle, and this way myotoxic effects of infiltration with such drugs are avoided. Myotoxicity is strongly related to the concentration of anesthetic injected, especially by long-lasting anesthetics. The use of epinephrine in concentrations of 1:100,000 or more can increase muscle damage caused local anesthetics. The myotoxicity of the botulinum toxin type-A is irreversible by binding to the presynaptic cholinergic nerve terminals and interrupting any neurogenic contraction of muscle fibers mediated by the affected motor plates. This chemical denervation maintains the paralysis of the muscle for a period of 3 to 6 months until new axons sprout from a motor nerve and form new synaptic contacts to restore a normal neuromuscular junction function of affected muscle fibers (17).

For the treatment of symptoms associated with TPs, the DDN is a technique as effective as the infiltration of anesthetics if it causes local twitch response, which occurs when the needle had inserted in the muscular active loci of the TP. The reverse is also true and, if there is no local twitch response, the DDN or the infiltration of anesthetics are equally ineffective. Another reason to prefer the DDN before using local anesthetics is because dry needling allows the location of all TPs from one area by preserving pain reaction to palpation.

The results of our study have shown that the greatest reduction in the magnitude of pain was achieved when patients had started from a more unfavorable clinical situation with intense pain, so it was expected that pain improvement would be more evident in these patients. We have observed that in those patients who had significant pain before starting treatment (values 8 to 10 in visual analog scale), it was common that they had a reduction of 6 points, while those that started with mild pain (value less than 6) the expected reduction of pain was 4 points or less. These findings are consistent with the results published by other authors that performed intramuscular infiltrations after muscle needling (18,19). Fernández-Carnero et al. conducted a study with DDN in masseter muscle TPs in a group of 12 women obtaining a good therapeutic outcome measured by increase in the threshold of pain caused by pressure and measured with algometer (15). Bubnov examined the DDN guided by ultrasound to increase the accuracy of the TP needling under visual verification, and obtained a pain reduction in 93.3% of patients in a group of 91 patients with myofascial pain in different locations (20).

In conclusion, the results of our study suggest that patients with painful temporomandibular disease due to external pterygoid muscle involvement treated selectively with needles for dry needling showed a significant improvement in pain and, as consequence, an improvement of functional limitation which persisted up to 6 months after finishing the treatment. Pain reduction was greater the higher was the intensity of pain at baseline.
